# Supplementary material for: Meta-2OM: A multi-classifier meta-model for the accurate prediction of RNA 2′-O-methylation sites in human RNA
Source: PLoS One. 2024 Jun 26;19(6):e0305406. doi: 10.1371/journal.pone.0305406 (PMC11207182; doi:10.1371/journal.pone.0305406)
Supplement: S1 Table — (PDF) [file pone.0305406.s001.pdf]

SUPPLEMENTAL INFORMATION

Table S1. Hyperparameter tuning for each machine-learning method.

ML	Hyperparameter	Search region	Optimal value
LGBM	boosting_type		gbdt
	metric		auc
	learning_rate	[0.01, 0.1, 0.2, 0.4]	0.2
	max_depth	[3,5,7]	5
	num_leaves	[5, 10, 20, 30]	5
	min_data_in_leaf	[10, 15, 20]	15
	min_child_samples	[5, 10, 20]	5
	num_iteration	[10, 20, 50, 100]	100
XGB	booster		gbtree
	objective		binary:logistic
	eval_metric		auc
	n_estimators	[10, 100, 500, 1000]	100
	learning_rate	[0.01, 0.1, 0.2, 0.4]	0.1
	max_depth	[2, 4, 6]	4
RF	n_estimators	[10, 100, 500, 1000]	500
	max_depth	[3, 5, 7, 9]	9
CBC	eval_metric		AUC
	loss_function		Logloss
	learning_rate	[0.01, 0.1, 0.2, 0.4, 0.5]	0.01
	depth	[5,6,7,8,9,12,16]	9
	iterations	[10,30,50,70,100,500]	500
SVM	kernel	-	rbf
	gamma	[1, 0.1, 0.01, 0.001, 0.0001]	0.1
	C	[0.1, 1, 5, 10, 15, 100, 1000]	1
NB	var_smoothing	[1e-2, 1e-3, 1e-4, 1e-5, 1e-6, 1e-7, 1e-8, 1e-9, 1e-10, 1e-11, 1e-12, 1e-13, 1e-14, 1e-15]	1e-2 = 0.01

The hyperparameters were optimized during the training by using GridSearchCV in scikit-learn. Default values were given for the parameter values not listed in the table.
