# Supplementary material for: Meta-2OM: A multi-classifier meta-model for the accurate prediction of RNA 2′-O-methylation sites in human RNA
Source: PLoS One. 2024 Jun 26;19(6):e0305406. doi: 10.1371/journal.pone.0305406 (PMC11207182; doi:10.1371/journal.pone.0305406)
Supplement: S2 Table — (PDF) [file pone.0305406.s002.pdf]

Table S2. Sequential performance of 144 baseline classifiers through the training datasets.

Rank	MLs	Encode	SEN	SPE	PRE	ACC	MCC	F1	AUC	AUPRC
1	LGBM	W2V	0.829	0.891	0.884	0.860	0.722	0.856	0.934	0.943
2	SVM	W2V	0.804	0.901	0.892	0.852	0.710	0.845	0.929	0.937
3	LR	W2V	0.805	0.880	0.872	0.842	0.689	0.836	0.916	0.926
4	LGBM	NPPS	0.767	0.905	0.890	0.836	0.679	0.824	0.915	0.926
5	CBC	W2V	0.793	0.869	0.858	0.831	0.664	0.824	0.912	0.923
6	LGBM	binary	0.763	0.906	0.892	0.834	0.677	0.821	0.912	0.925
7	LGBM	EIIP	0.759	0.901	0.885	0.830	0.667	0.817	0.907	0.921
8	LGBM	BPB	0.731	0.920	0.902	0.826	0.664	0.807	0.906	0.920
9	CBC	NPPS	0.751	0.886	0.869	0.818	0.644	0.805	0.900	0.915
10	SVM	Kmer	0.805	0.839	0.834	0.822	0.645	0.819	0.898	0.900
11	LGBM	NCP	0.752	0.884	0.868	0.818	0.643	0.804	0.898	0.913
12	SVM	NCP-ND	0.747	0.884	0.867	0.816	0.638	0.801	0.893	0.908
13	LGBM	Kmer	0.805	0.824	0.824	0.815	0.632	0.813	0.892	0.894
14	LGBM	NCP-ND	0.728	0.888	0.870	0.808	0.626	0.791	0.890	0.906
15	SVM	NCP	0.700	0.919	0.899	0.810	0.637	0.785	0.890	0.906
16	SVM	binary	0.703	0.918	0.898	0.811	0.638	0.787	0.890	0.906
17	SVM	CKSNAP	0.799	0.824	0.820	0.811	0.624	0.809	0.888	0.888
18	SVM	NPS	0.821	0.795	0.802	0.808	0.618	0.810	0.887	0.886
19	CBC	Kmer	0.762	0.844	0.834	0.803	0.611	0.794	0.885	0.890
20	SVM	NPPS	0.727	0.888	0.867	0.807	0.623	0.790	0.884	0.902
21	LGBM	ENAC	0.700	0.891	0.871	0.796	0.606	0.772	0.884	0.899
22	SVM	TNC	0.775	0.836	0.827	0.806	0.614	0.799	0.883	0.884
23	SVM	PseEIIP	0.800	0.809	0.811	0.804	0.612	0.803	0.882	0.883
24	CBC	binary	0.716	0.878	0.856	0.797	0.603	0.779	0.882	0.900
25	SVM	ENAC	0.714	0.886	0.864	0.800	0.610	0.780	0.881	0.894
26	CBC	NPS	0.784	0.817	0.815	0.800	0.605	0.797	0.880	0.879
27	LGBM	CKSNAP	0.781	0.821	0.816	0.801	0.605	0.797	0.880	0.880
28	LGBM	NPS	0.810	0.800	0.803	0.805	0.611	0.806	0.880	0.879
29	CBC	CKSNAP	0.799	0.801	0.805	0.800	0.604	0.800	0.879	0.878
30	CBC	BPB	0.665	0.917	0.892	0.791	0.602	0.760	0.879	0.898
31	CBC	PseEIIP	0.789	0.813	0.813	0.801	0.607	0.798	0.879	0.881
32	CBC	TNC	0.789	0.813	0.813	0.801	0.607	0.798	0.879	0.881
33	LGBM	PseEIIP	0.764	0.829	0.821	0.797	0.598	0.789	0.878	0.878
34	LGBM	TNC	0.764	0.829	0.821	0.797	0.598	0.789	0.878	0.878
35	CBC	NCP	0.683	0.902	0.877	0.793	0.601	0.767	0.877	0.897
36	CBC	PseKNC	0.811	0.796	0.800	0.804	0.608	0.805	0.877	0.878
37	LGBM	PseKNC	0.776	0.815	0.810	0.795	0.594	0.791	0.876	0.877
38	CBC	NCP-ND	0.663	0.915	0.886	0.789	0.597	0.758	0.875	0.894
39	LR	CKSNAP	0.796	0.795	0.797	0.796	0.593	0.796	0.874	0.875
40	CBC	EIIP	0.662	0.911	0.882	0.786	0.592	0.756	0.873	0.893
41	LR	NPPS	0.672	0.912	0.886	0.792	0.603	0.763	0.873	0.894
42	LR	NPS	0.785	0.800	0.801	0.792	0.589	0.790	0.871	0.872
43	CBC	DNC	0.769	0.807	0.802	0.788	0.579	0.784	0.867	0.863

44	SVM	DNC	0.787	0.792	0.793	0.790	0.582	0.788	0.866	0.857
45	SVM	PseKNC	0.802	0.776	0.784	0.789	0.580	0.792	0.865	0.864
46	CBC	ENAC	0.690	0.864	0.840	0.777	0.566	0.754	0.864	0.880
47	LR	Kmer	0.751	0.822	0.810	0.786	0.576	0.778	0.864	0.866
48	LGBM	DNC	0.771	0.807	0.801	0.789	0.579	0.785	0.864	0.860
49	LR	TNC	0.772	0.807	0.800	0.789	0.579	0.786	0.861	0.859
50	RF	W2V	0.622	0.913	0.888	0.768	0.566	0.726	0.861	0.878
51	LR	NCP-ND	0.692	0.871	0.845	0.782	0.573	0.760	0.859	0.874
52	RF	NPPS	0.596	0.940	0.912	0.768	0.573	0.718	0.858	0.882
53	LGBM	CTD	0.782	0.768	0.773	0.775	0.552	0.776	0.855	0.858
54	XGB	NPPS	0.630	0.911	0.880	0.771	0.566	0.733	0.852	0.875
55	XGB	W2V	0.600	0.933	0.903	0.767	0.567	0.719	0.849	0.874
56	RF	TNC	0.761	0.789	0.784	0.775	0.551	0.772	0.849	0.848
57	RF	PseEIIP	0.761	0.789	0.784	0.775	0.551	0.772	0.849	0.848
58	RF	PseKNC	0.775	0.773	0.774	0.774	0.548	0.774	0.848	0.844
59	XGB	CKSNAP	0.768	0.784	0.783	0.776	0.554	0.773	0.846	0.842
60	XGB	NPS	0.765	0.786	0.785	0.776	0.554	0.772	0.846	0.841
61	NB	Kmer	0.788	0.766	0.772	0.777	0.555	0.780	0.846	0.848
62	RF	Kmer	0.654	0.870	0.839	0.762	0.540	0.732	0.845	0.856
63	LR	DNC	0.799	0.741	0.756	0.770	0.542	0.776	0.845	0.835
64	KN	W2V	0.667	0.880	0.852	0.773	0.563	0.743	0.844	0.866
65	CBC	CTD	0.704	0.820	0.797	0.762	0.528	0.747	0.844	0.846
66	NB	NPPS	0.602	0.928	0.894	0.765	0.562	0.720	0.844	0.873
67	RF	DNC	0.799	0.746	0.760	0.772	0.546	0.778	0.842	0.832
68	XGB	DNC	0.753	0.786	0.781	0.769	0.542	0.765	0.840	0.832
69	NB	PseEIIP	0.802	0.737	0.754	0.770	0.541	0.777	0.840	0.826
70	NB	TNC	0.802	0.737	0.754	0.770	0.541	0.777	0.840	0.826
71	LR	binary	0.643	0.891	0.857	0.767	0.552	0.733	0.840	0.861
72	LR	NCP	0.643	0.891	0.857	0.767	0.552	0.733	0.840	0.861
73	XGB	PseKNC	0.768	0.759	0.765	0.763	0.531	0.763	0.840	0.834
74	SVM	EIIP	0.642	0.881	0.844	0.762	0.539	0.729	0.839	0.856
75	SVM	BPB	0.552	0.970	0.948	0.761	0.574	0.697	0.839	0.872
76	KN	binary	0.653	0.879	0.844	0.766	0.546	0.736	0.836	0.860
77	KN	NCP	0.653	0.879	0.844	0.766	0.546	0.736	0.836	0.860
78	KN	NPPS	0.609	0.930	0.902	0.770	0.572	0.725	0.835	0.871
79	RF	NPS	0.795	0.720	0.743	0.757	0.520	0.765	0.834	0.828
80	RF	NCP-ND	0.560	0.950	0.919	0.755	0.555	0.696	0.833	0.862
81	XGB	TNC	0.780	0.738	0.750	0.759	0.520	0.764	0.833	0.832
82	XGB	PseEIIP	0.780	0.738	0.750	0.759	0.520	0.764	0.833	0.832
83	LR	PseEIIP	0.755	0.763	0.763	0.759	0.520	0.758	0.832	0.826
84	NB	PseKNC	0.789	0.729	0.746	0.759	0.521	0.765	0.832	0.818
85	LR	ENAC	0.627	0.892	0.856	0.759	0.539	0.722	0.831	0.852
86	KN	NCP-ND	0.680	0.848	0.818	0.764	0.536	0.743	0.831	0.853
87	NB	W2V	0.690	0.832	0.804	0.761	0.528	0.743	0.826	0.846
88	CBC	RCKmer	0.767	0.737	0.747	0.752	0.506	0.756	0.825	0.818
89	RF	CKSNAP	0.763	0.733	0.743	0.748	0.499	0.751	0.824	0.818

90	SVM	RCKmer	0.737	0.765	0.758	0.751	0.502	0.748	0.823	0.811
91	LGBM	RCKmer	0.765	0.730	0.740	0.748	0.496	0.752	0.821	0.813
92	SVM	CTD	0.712	0.776	0.765	0.744	0.492	0.734	0.820	0.818
93	RF	BPB	0.552	0.954	0.925	0.753	0.554	0.691	0.819	0.857
94	NB	DNC	0.749	0.752	0.752	0.751	0.502	0.750	0.819	0.801
95	LR	BPB	0.560	0.952	0.922	0.756	0.557	0.695	0.818	0.856
96	NB	binary	0.579	0.929	0.891	0.754	0.543	0.702	0.818	0.851
97	XGB	NCP-ND	0.564	0.920	0.876	0.742	0.518	0.686	0.817	0.843
98	RF	binary	0.536	0.965	0.940	0.751	0.556	0.682	0.816	0.855
99	RF	NCP	0.545	0.950	0.917	0.747	0.542	0.682	0.816	0.852
100	LR	CTD	0.714	0.768	0.759	0.741	0.486	0.734	0.815	0.811
101	XGB	Kmer	0.656	0.813	0.788	0.734	0.483	0.709	0.815	0.826
102	NB	NCP-ND	0.671	0.809	0.782	0.740	0.487	0.720	0.814	0.838
103	NB	NCP	0.556	0.958	0.931	0.757	0.562	0.696	0.814	0.853
104	XGB	binary	0.558	0.920	0.878	0.739	0.515	0.680	0.814	0.844
105	NB	BPB	0.544	0.953	0.921	0.749	0.545	0.684	0.814	0.851
106	XGB	BPB	0.498	0.960	0.927	0.729	0.517	0.647	0.813	0.844
107	RF	ENAC	0.545	0.903	0.850	0.724	0.480	0.664	0.813	0.833
108	KN	Kmer	0.753	0.740	0.744	0.747	0.494	0.749	0.812	0.818
109	RF	EIIP	0.558	0.940	0.904	0.749	0.539	0.690	0.811	0.846
110	XGB	NCP	0.539	0.937	0.897	0.738	0.519	0.672	0.811	0.841
111	RF	RCKmer	0.738	0.737	0.743	0.737	0.480	0.736	0.810	0.798
112	KN	TNC	0.759	0.733	0.740	0.746	0.492	0.749	0.809	0.814
113	XGB	EIIP	0.521	0.953	0.918	0.737	0.526	0.664	0.809	0.841
114	XGB	RCKmer	0.788	0.684	0.714	0.736	0.474	0.749	0.808	0.801
115	XGB	ENAC	0.547	0.893	0.841	0.720	0.471	0.660	0.807	0.826
116	LR	RCKmer	0.777	0.689	0.716	0.733	0.470	0.744	0.805	0.793
117	NB	ENAC	0.676	0.793	0.766	0.734	0.472	0.718	0.804	0.812
118	KN	PseEIIP	0.724	0.751	0.750	0.737	0.479	0.732	0.804	0.807
119	XGB	CTD	0.737	0.723	0.728	0.730	0.461	0.731	0.802	0.795
120	KN	DNC	0.753	0.729	0.736	0.741	0.483	0.744	0.802	0.802
121	RF	CTD	0.776	0.672	0.706	0.724	0.453	0.737	0.801	0.797
122	KN	NPS	0.760	0.709	0.723	0.734	0.470	0.741	0.799	0.802
123	LR	EIIP	0.572	0.881	0.831	0.727	0.478	0.676	0.798	0.800
124	LR	PseKNC	0.836	0.609	0.682	0.722	0.457	0.751	0.798	0.770
125	KN	CKSNAP	0.764	0.696	0.716	0.730	0.461	0.739	0.798	0.799
126	KN	PseKNC	0.751	0.706	0.724	0.729	0.463	0.733	0.794	0.795
127	NB	EIIP	0.583	0.866	0.816	0.725	0.470	0.678	0.793	0.791
128	NB	NPS	0.752	0.707	0.722	0.730	0.462	0.735	0.793	0.777
129	KN	ENAC	0.674	0.784	0.757	0.729	0.460	0.713	0.793	0.811
130	KN	BPB	0.526	0.953	0.920	0.739	0.530	0.667	0.788	0.836
131	NB	CTD	0.709	0.727	0.727	0.718	0.440	0.714	0.786	0.769
132	NB	CKSNAP	0.746	0.698	0.713	0.722	0.446	0.728	0.784	0.766
133	NB	RCKmer	0.763	0.655	0.694	0.709	0.428	0.723	0.783	0.764
134	KN	EIIP	0.590	0.820	0.767	0.705	0.422	0.667	0.764	0.784
135	KN	RCKmer	0.708	0.706	0.707	0.707	0.414	0.708	0.759	0.758

136	KN	CTD	0.618	0.735	0.709	0.676	0.364	0.651	0.740	0.748
137	CBC	ANF	0.604	0.707	0.689	0.655	0.323	0.627	0.721	0.721
138	LGBM	ANF	0.721	0.584	0.641	0.652	0.317	0.671	0.716	0.714
139	SVM	ANF	0.634	0.653	0.655	0.644	0.295	0.635	0.694	0.681
140	LR	ANF	0.709	0.556	0.620	0.633	0.274	0.657	0.685	0.667
141	RF	ANF	0.793	0.434	0.584	0.614	0.244	0.672	0.667	0.648
142	XGB	ANF	0.828	0.388	0.576	0.608	0.246	0.678	0.661	0.650
143	NB	ANF	0.734	0.490	0.593	0.612	0.238	0.652	0.647	0.618
144	KN	ANF	0.836	0.252	0.529	0.544	0.115	0.644	0.565	0.573
