# Supplementary material for: Meta-2OM: A multi-classifier meta-model for the accurate prediction of RNA 2′-O-methylation sites in human RNA
Source: PLoS One. 2024 Jun 26;19(6):e0305406. doi: 10.1371/journal.pone.0305406 (PMC11207182; doi:10.1371/journal.pone.0305406)
Supplement: S3 Table — (PDF) [file pone.0305406.s003.pdf]

Table S3. The individual performance of the nucleotide-specific model and a comparison with the generic model under training and test datasets.

Meta-classifier	Train					Test				
	SEN	SPE	ACC	MCC	AUC	SEN	SPE	ACC	MCC	AUC
Am	0.913	0.948	0.931	0.863	0.975	0.850	0.904	0.877	0.755	0.931
Cm	0.868	0.952	0.910	0.824	0.964	0.822	0.911	0.866	0.736	0.938
Gm	0.906	0.927	0.916	0.834	0.969	0.844	0.891	0.868	0.737	0.936
Um	0.849	0.865	0.857	0.724	0.938	0.783	0.806	0.795	0.597	0.889
Mean {(Am+Cm+Gm+Um)/4}	**0.884**	**0.923**	**0.904**	**0.811**	**0.961**	0.825	0.878	0.851	0.706	0.923
Generic (Nm)	0.871	0.918	0.894	0.791	0.958	**0.836**	**0.904**	**0.870**	**0.743**	**0.940**

Bold indicates the highest score for the corresponding statistical measures
