# Supplementary material for: Meta-2OM: A multi-classifier meta-model for the accurate prediction of RNA 2′-O-methylation sites in human RNA
Source: PLoS One. 2024 Jun 26;19(6):e0305406. doi: 10.1371/journal.pone.0305406 (PMC11207182; doi:10.1371/journal.pone.0305406)
Supplement: S1 File — Also, the stacking performance representation is based on nucleotide-specific datasets. (PDF) [file pone.0305406.s004.pdf]

SUPPLEMENTAL INFORMATION

Supplementary Figures

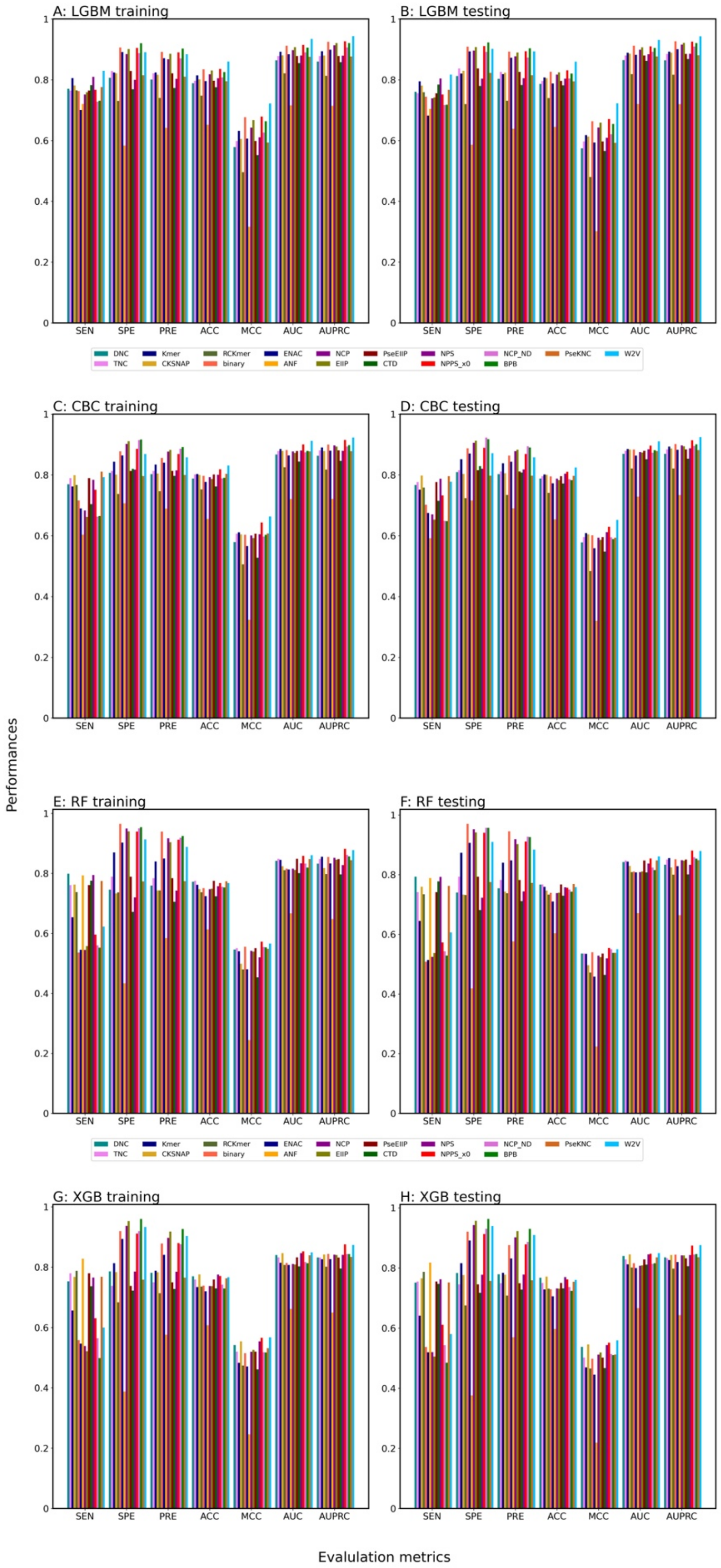

Fig S1. The performance analysis of LGBM, CBC, RF, and XGB to predict the Nm sites under 18 different encoding models.

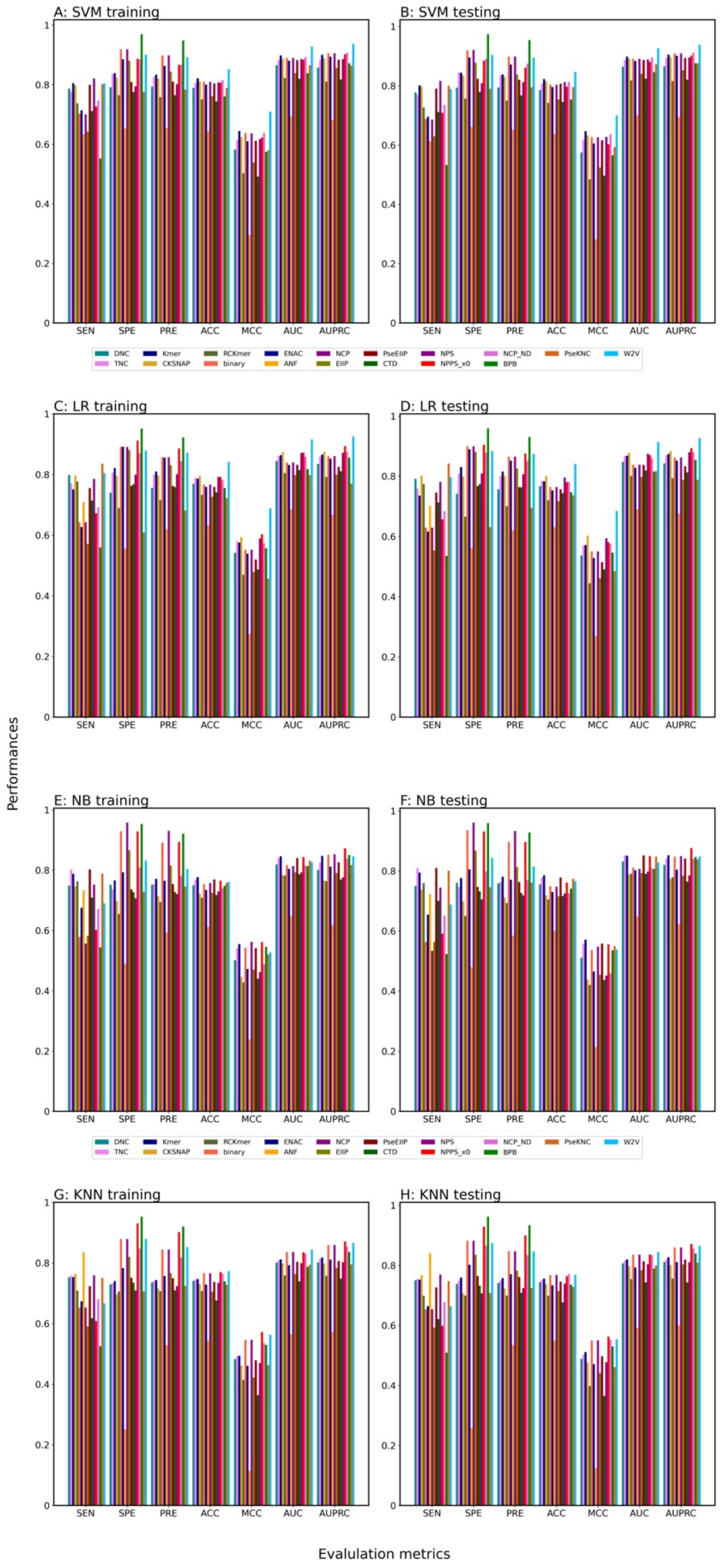

Fig S2. The performance analysis of SVM, LR, NB, and KNN to predict the Nm sites under 18 different encoding models.

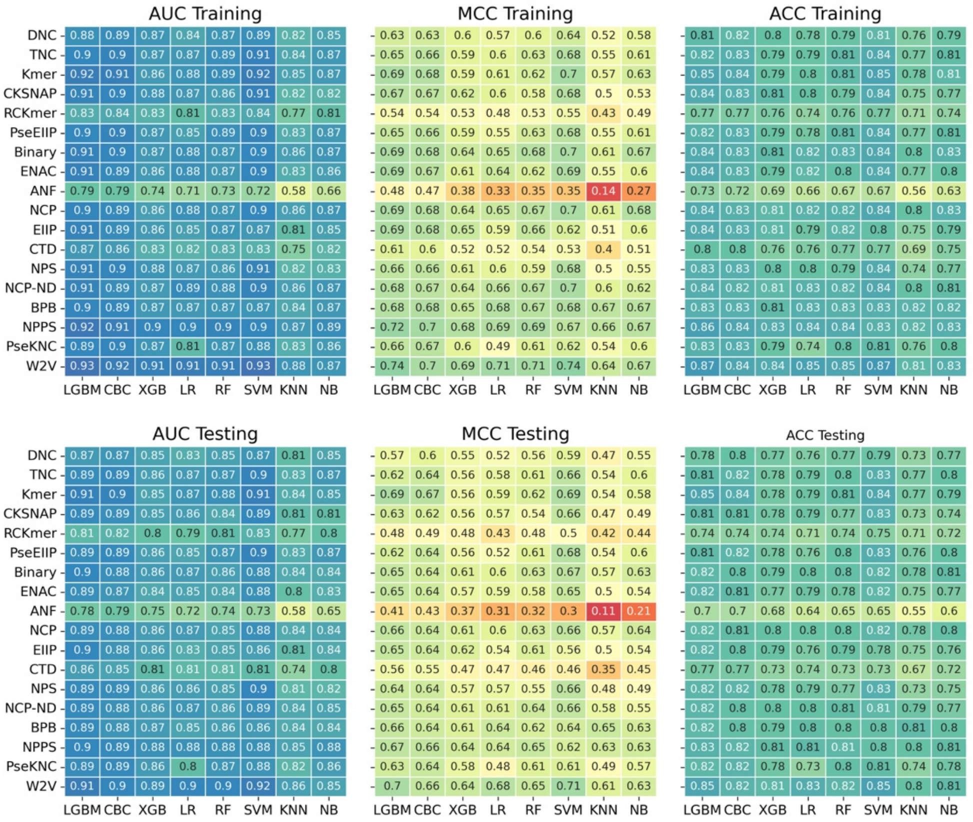

Fig S3. Performance analysis of the baseline classifiers under nucleotide-specific Am datasets. The classifiers were generated through eight different MLs with eighteen single-feature encoding methods. The MCC, ACC, and AUC are presented on the training (A, B, C) and independent (D, E, F) datasets.

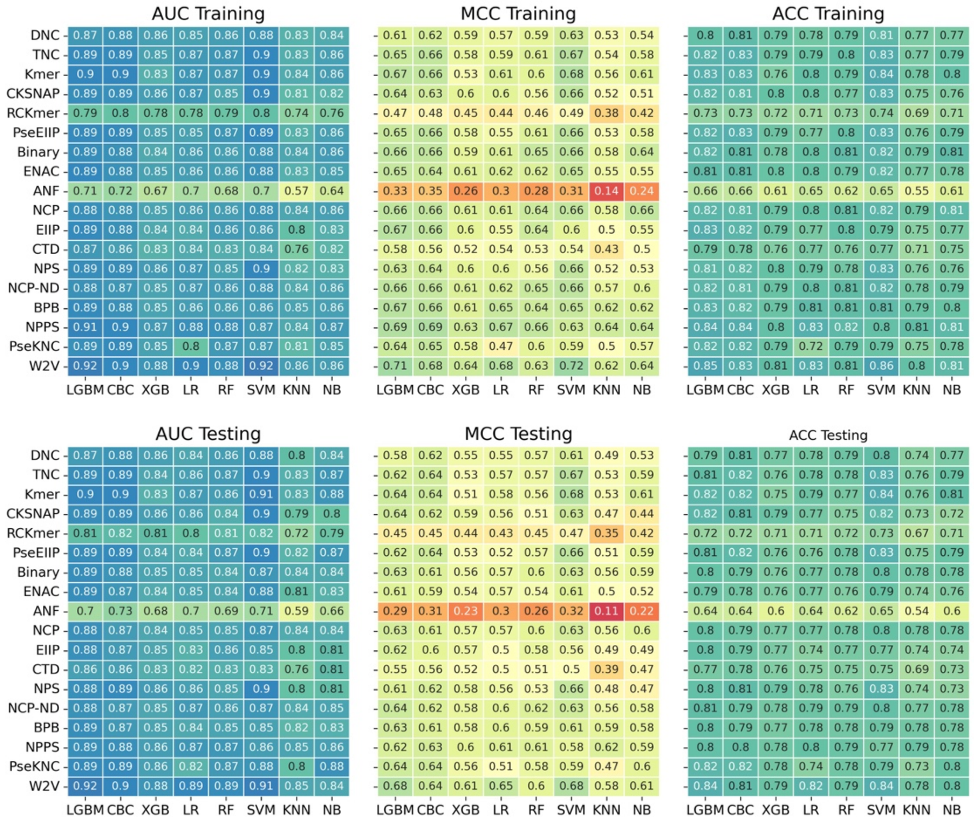

Fig S4. Performance analysis of the baseline classifiers under nucleotide-specific Cm datasets. The classifiers were generated through eight different MLs with eighteen single-feature encoding methods. The MCC, ACC, and AUC are presented on the training (A, B, C) and independent (D, E, F) datasets.

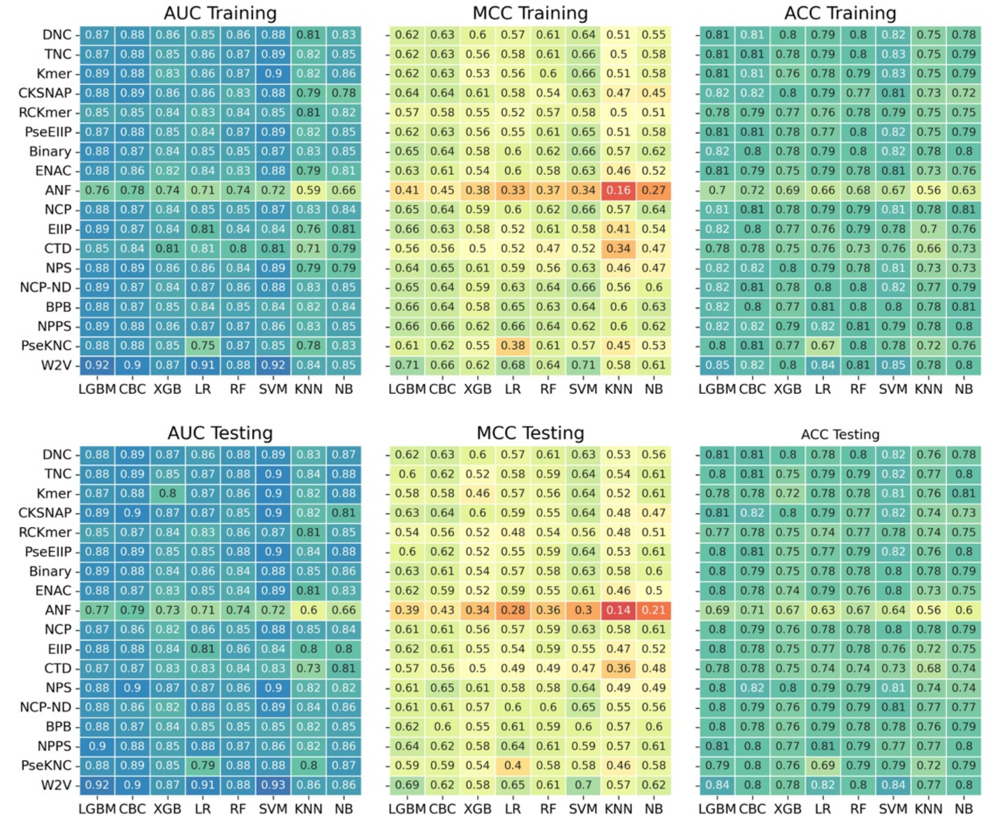

Fig S5. Performance analysis of the baseline classifiers under nucleotide-specific Gm datasets. The classifiers were generated through eight different MLs with eighteen single-feature encoding methods. The MCC, ACC, and AUC are presented on the training (A, B, C) and independent (D, E, F) datasets.

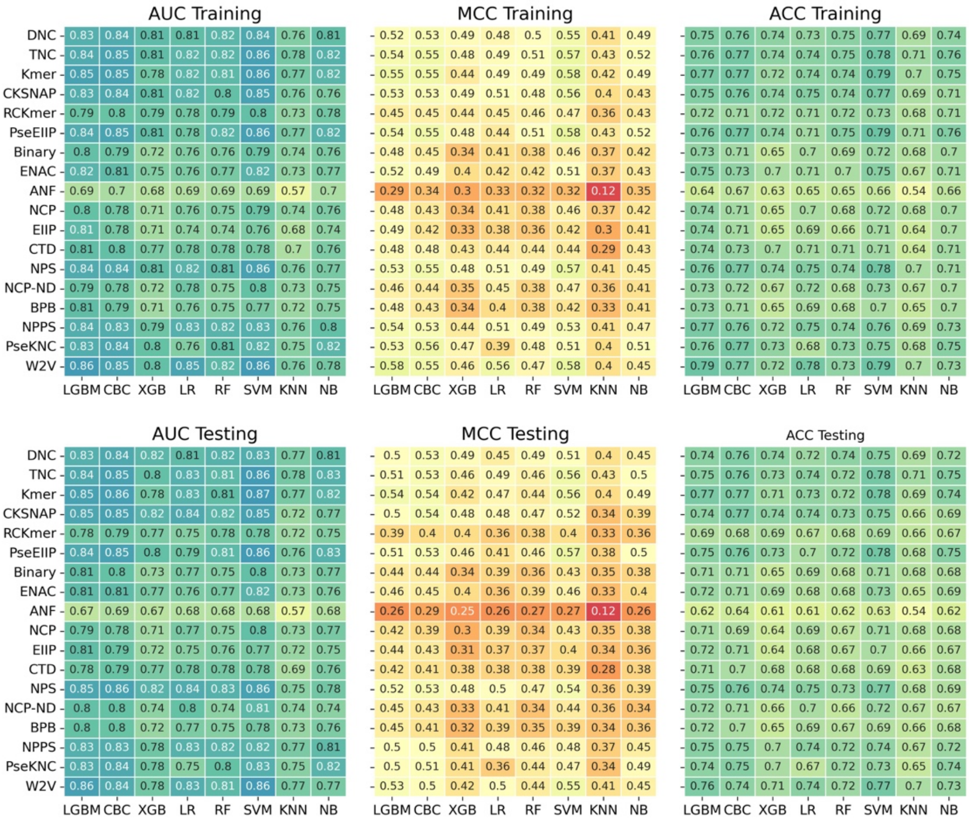

Fig S6. Performance analysis of the baseline classifiers under nucleotide-specific Um datasets. The classifiers were generated through eight different MLs with eighteen single-feature encoding methods. The MCC, ACC, and AUC are presented on the training (A, B, C) and independent (D, E, F) datasets.

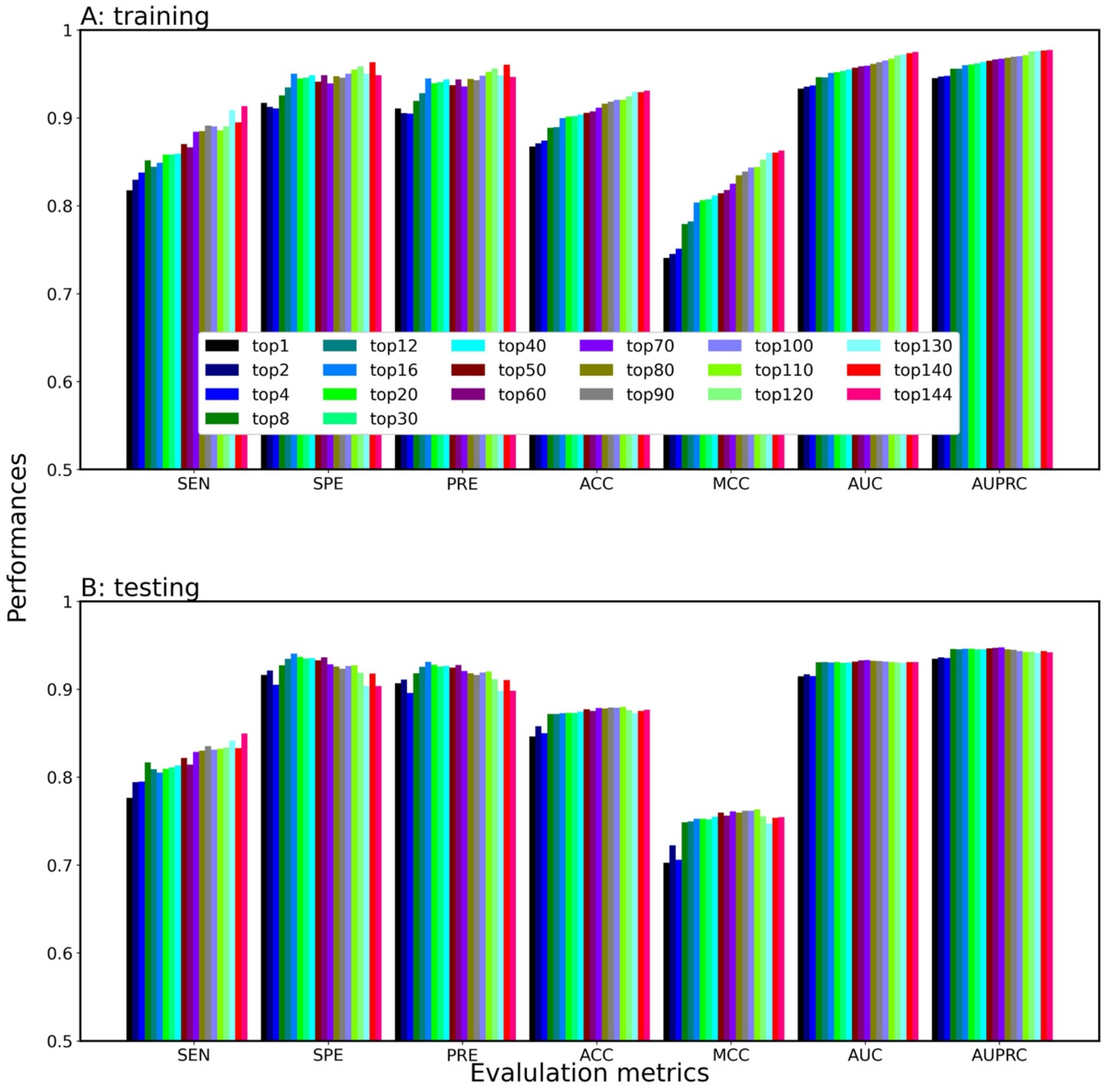

Fig S7. Effect of the employed feature sets on meta-classifiers under nucleotide-specific Am datasets. The 20 meta-classifiers performance was compared based on (A) training and (B) independent datasets.

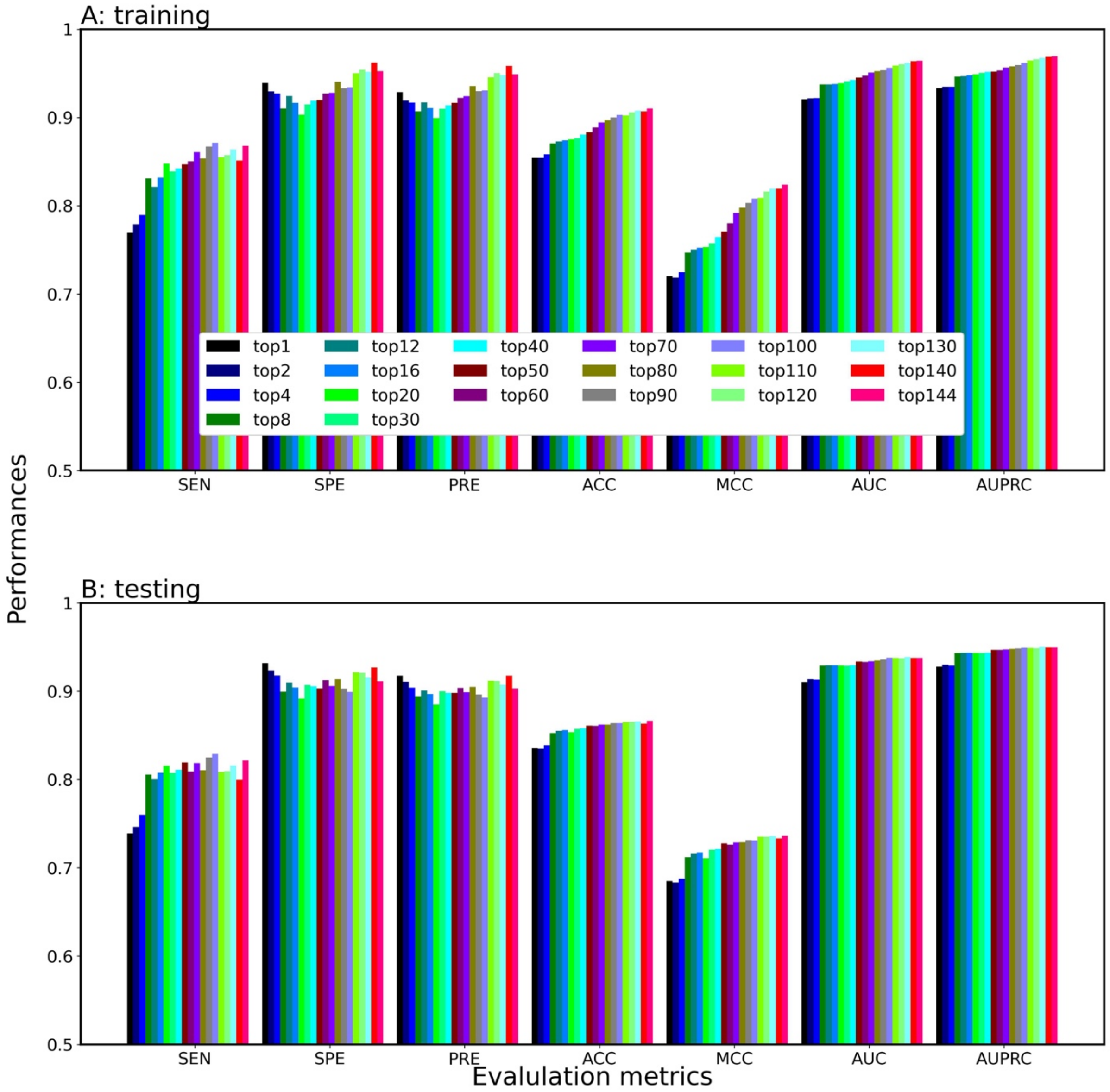

Fig S8. Effect of the employed feature sets on meta-classifiers under nucleotide-specific Cm datasets. The 20 meta-classifiers performance was compared based on (A) training and (B) independent datasets.

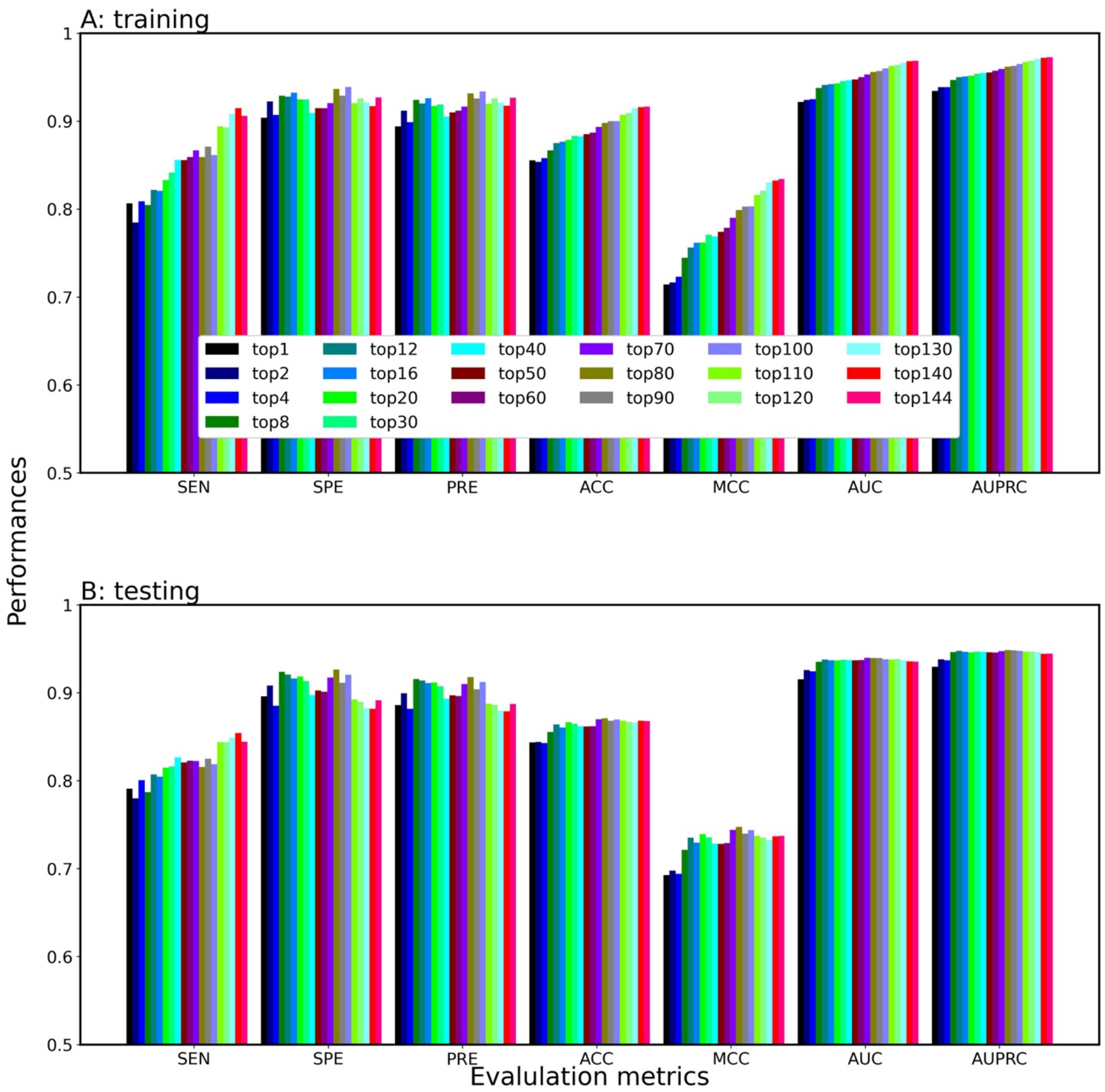

Fig S9. Effect of the employed feature sets on meta-classifiers under nucleotide-specific Gm datasets. The 20 meta-classifiers performance was compared based on (A) training and (B) independent datasets.

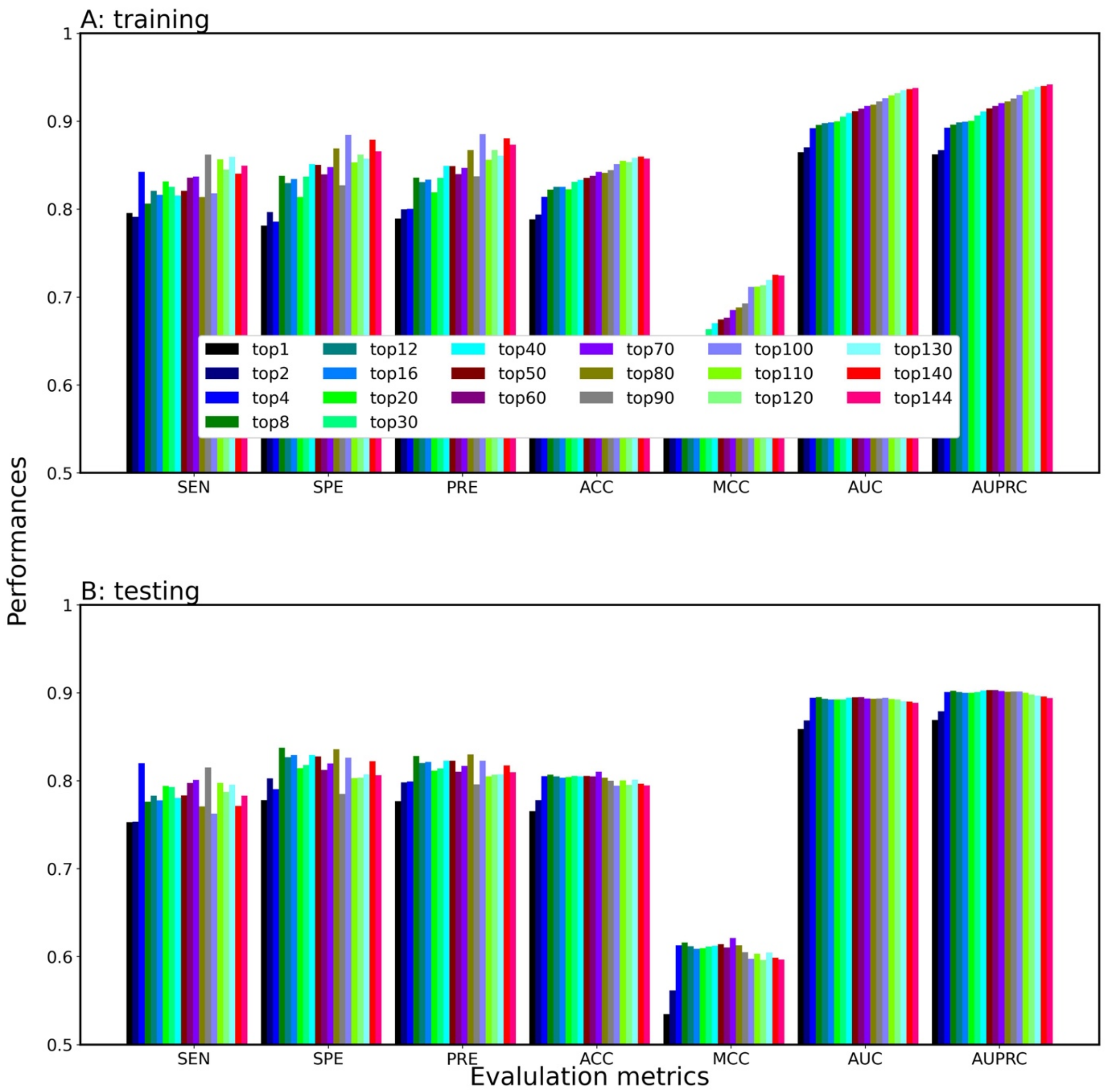

Fig S10. Effect of the employed feature sets on meta-classifiers under nucleotide-specific Am datasets. The 20 meta-classifiers performance was compared based on (A) training and (B) independent datasets.
